# Cell-Free Hemoglobin Concentration in Blood Prime Solution Is a Major Determinant of Cell-Free Hemoglobin Exposure during Cardiopulmonary Bypass Circulation in the Newborn

**DOI:** 10.3390/jcm11144071

**Published:** 2022-07-14

**Authors:** Åsa Jungner, Suvi Vallius, Magnus Gram, David Ley

**Affiliations:** 1 Pediatrics Lund, Department of Clinical Sciences Lund, Skåne University Hospital, Lund University, 222 41 Lund, Sweden; david.ley@med.lu.se; 2 Pediatrics Lund, Department of Clinical Sciences Lund, Lund University, 221 85 Lund, Sweden; suvi.vallius@med.lu.se (S.V.); magnus.gram@med.lu.se (M.G.)

**Keywords:** cell-free hemoglobin, cardiopulmonary bypass, congenital heart defect, neonate, haptoglobin, hemopexin

## Abstract

Exposure to circulating cell-free hemoglobin is a ubiquitous feature of open-heart surgery on cardiopulmonary bypass circulation. This study aims to determine the origins and dynamics of circulating cell-free hemoglobin and its major scavenger proteins haptoglobin and hemopexin during neonatal cardiopulmonary bypass. Forty neonates with an isolated critical congenital heart defect were included in a single-center prospective observational study. Blood samples were obtained preoperatively, hourly during bypass circulation, after bypass separation, at admission to the pediatric intensive care unit, and at postoperative days 1–3. Concentrations of cell-free hemoglobin, haptoglobin and hemopexin were determined using ELISA. Neonates were exposed to significantly elevated plasma concentrations of cell-free hemoglobin and a concomitant depletion of scavenger protein supplies during open-heart surgery. The main predictor of cell-free hemoglobin exposure was the concentration of cell-free hemoglobin in blood prime solution. Concentrations of haptoglobin and hemopexin in prime solution were important determinants for intra- and postoperative circulating scavenger protein resources.

## 1. Introduction

Cell-free hemoglobin (Hb) is a toxic molecule with oxidative, endothelial disrupting and proinflammatory properties [1,2,3,4]. During neonatal cardiopulmonary bypass (CPB) circulation, the newborn is exposed to increased levels of cell-free hemoglobin and concomitantly to depleted levels of hemoglobin-scavenging proteins [5,6]. Prior research states that increased concentrations of cell-free hemoglobin during pediatric cardiac surgery are associated with an increased risk of postoperative acute kidney injury [7,8]. Although circulating cell-free hemoglobin is implicated in organ damage, investigations of the origin and dynamics of cell-free hemoglobin exposure during neonatal open-heart surgery are scarce.

This article reports on determinants of cell-free hemoglobin exposure and its primary scavenger proteins haptoglobin and hemopexin during cardiopulmonary bypass circulation in newborns with an isolated critical congenital heart defect (cCHD). The present investigation is part of a larger, not yet published, prospective observational clinical study focusing on evaluating the impact of cell-free hemoglobin exposure and supranormal oxygen tensions on postoperative white matter brain integrity. We hypothesized that the neonates’ requirement of a blood primed bypass circuit would introduce additional determinants of cell-free hemoglobin exposure other than the red blood cell (RBC) trauma usually considered during bypass circulation.

## 2. Materials and Methods

### 2.1. Study Participants

Forty term newborns with an isolated cCHD requiring open-heart surgery on CPB circulation within 30 days of life were recruited to the prospective observational clinical study. Newborns with multiple malformations, confirmed or suspected syndrome diagnosis, perinatal asphyxia defined as hypoxic-ischemic encephalopathy grades 2–3 or severe preoperative hemodynamic instability requiring treatment on extracorporeal membrane oxygenation were not considered eligible. Clinical characteristics of included neonates are presented in Table 1.

### 2.2. Sampling Regimen

Preoperative sampling (denoted pre) was obtained before induction of anesthesia on the day of surgery in neonates with a preoperative arterial line (*n* = 38). In neonates without a preoperative arterial line (*n* = 2), sampling was obtained after induction of anesthesia and placement of an arterial line. Intraoperative sampling was attained after bypass start (denoted t00), hourly from arterial and venous cannulas, respectively, when on bypass circulation (denoted t01–04), and after bypass separation (denoted post). Postoperative sampling was obtained at admission to the pediatric intensive care unit (PICU) denoted postoperative day 0 (pod0), and in the morning of postoperative days 1–3 (pod1–3), respectively. Sampling was discontinued when the arterial line was withdrawn. Blood samples were immediately centrifuged, plasma separated and snap-frozen on dry ice. Samples were stored in −80 °C until analysis.

### 2.3. CPB Circuit Components and Flow

All neonates were operated using a Stöckert S5 Perfusion System (Sorin Group, München, Germany) equipped with roller pumps and a Capiox FX05 oxygenator (Terumo, Tokyo, Japan). Tubing size, cannula size and cannulation strategy were chosen according to the neonate’s size and planned surgery. Total body perfusion bypass flow was calculated to achieve 3.0 L/min/m${}^{2}$ for neonates weighting ≥ 3.0 kg, and 3.3 L/min/m${}^{2}$ for neonates weighting < 3.0 kg. For selective cerebral perfusion, bypass flow was reduced to 1/3 of the calculated flow during total body perfusion. RBC salvaging was performed after separation from bypass.

### 2.4. ELISA Analysis of Cell-Free Hemoglobin, Haptoglobin and Hemopexin

Circulating concentrations of cell-free hemoglobin, haptoglobin and hemopexin were measured with commercially available solid-phase enzyme-linked immunosorbent assay kits (Hemoglobin Human ELISA Kit; ab157707, Abcam, Cambridge, UK; Haptoglobin ELISA Kit (Human) OKIA00064, Aviva Systems Biology, San Diego, CA, USA and Hemopexin ELISA Kit (Human) OKIA00066, Aviva Systems Biology, San Diego, CA, USA) according to the manufacturer’s instructions.

### 2.5. Area under Curve Calculations of Cell-Free Hemoglobin and Oxygen Exposure

Exposure to cell-free hemoglobin was quantified as area under curve (AUC) calculated from plotting plasma concentrations of cell-free hemoglobin obtained from start of bypass circulation until the morning of pod1 or at the time of PICU admission according to the chosen analysis. Baseline for AUC calculations was set to *y* = 0, *x*-axis scale was set to hours and minutes with bypass start as *x* = 0.

**Figure 1:**
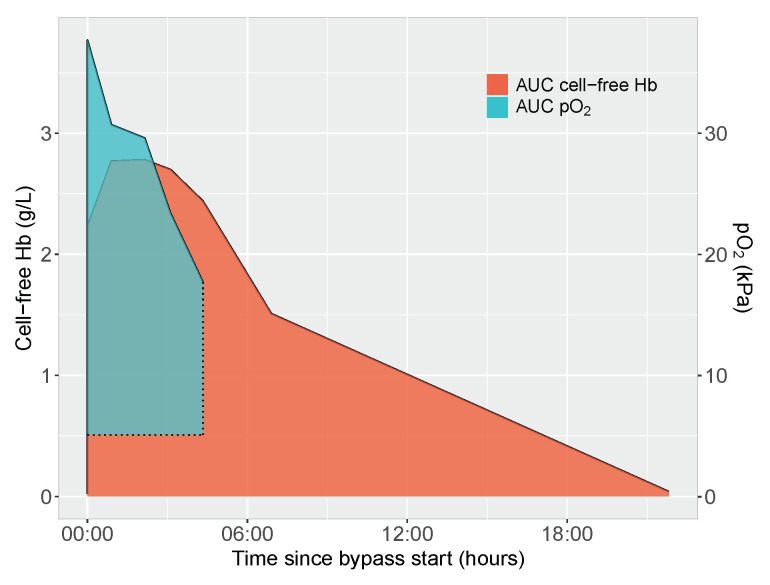
Representative image of area under curve (AUC) determinations for cell-free hemoglobin exposure (red) and oxygen exposure (turquoise) in one patient. Cell-free hemoglobin exposure was calculated as area under curve from start of bypass (*x* = 0) and terminated at postoperative day 1 or at PICU admission according to chosen analysis. Oxygen exposure was calculated as area under curve from start of bypass (*x* = 0) until separation of bypass. Baseline for calculations of oxygen exposure was set to preoperative oxygen tension. Cell-free hemoglobin concentrations are displayed on the left *y*-axis, whereas oxygen tensions are displayed on the right *y*-axis. Hb = hemoglobin, AUC = area under curve.

Oxygen exposure was quantified as AUC calculated from start of bypass until separation from bypass. Baseline for calculations was set to the preoperative oxygen tension. A representative image of AUC-calculations of cell-free hemoglobin exposure and oxygen exposure in one of the included infants is given in Figure 1.

### 2.6. Evaluated Clinical Determinants of Cell-Free Hemoglobin Exposure and Circulating Scavenger Protein Concentrations

The following variables were evaluated as potential predictors of cell-free hemoglobin exposure (AUC) in univariable and multivariable analysis: concentration of cell-free hemoglobin in blood prime solution (g/L), volume of RBCs in blood prime solution (mL/kg), storage length of RBC unit in prime solution (days), choice of CPB oxygenator and tubing, CPB flow (L/min/m${}^{2}$), time on CPB (minutes), continuous ultrafiltration (yes/no), vacuum-assisted venous drainage (yes/no), perfusionist’s notes on excessive suction (yes/no), transfusion of RBC during surgery (mL/kg) and oxygen exposure during CPB (AUC).

All neonates were operated using the same oxygenator and all but one used the same tubing sizes, rendering the two variables unsuitable for further analysis. All documented flow rates were in accordance with the calculated flow rates and varied little between patients, and CPB flow was thus discarded as an independent predictor for exposure to cell-free hemoglobin. Perfusionists’ notes on excessive suction were a rare event in our cohort (*n* = 2) and not further analyzed.

The following variables were evaluated as potential predictors of intra- and postoperative haptoglobin and hemopexin concentrations in univariable and multivariable analysis: preoperative endogenous plasma haptoglobin and hemopexin concentrations (g/L), volume of FFP in blood prime solution (mL/kg), haptoglobin and hemopexin concentrations in blood prime solution (g/L), volume of intraoperatively administered FFP (mL/kg), and exposure to cell-free hemoglobin (AUC) during surgery. All included neonates received intraoperative corticosteroids as per protocol. Details of potential clinical determinants are given in Table 2.

### 2.7. Statistical Considerations

Wilcoxon rank-sum test was used for groupwise comparisons of non-paired data with nonparametric distributions. Wilcoxon signed-rank test was used for groupwise comparisons of paired data with nonparametric distributions. Correlation between data with nonparametric distributions was calculated using Spearman’s rank correlation.

The selected clinical variables were first evaluated using univariable linear regression modeling and, if statistically significant, included in a subsequent multivariable linear regression model. For all models, no more than one independent variable per ten observations was allowed. Only models with acceptable residual diagnostics were acknowledged. *p*-values < 0.05 were considered significant. All statistical calculations and graphing were done using RStudio version 3.5.2.

## 3. Results

### 3.1. Determinants of Cell-Free Hemoglobin Exposure during Neonatal Cardiopulmonary Bypass Surgery

All neonates received a blood prime solution as per protocol. The packed RBC volume added to the circuit varied between 29–65 mL/kg, aiming for a hematocrit after bypass start of 0.3–0.35. Cell-free hemoglobin median concentration in blood prime solution was 0.51 g/L (interquartile range (IQR) 0.31–0.97).

**Figure 2:**
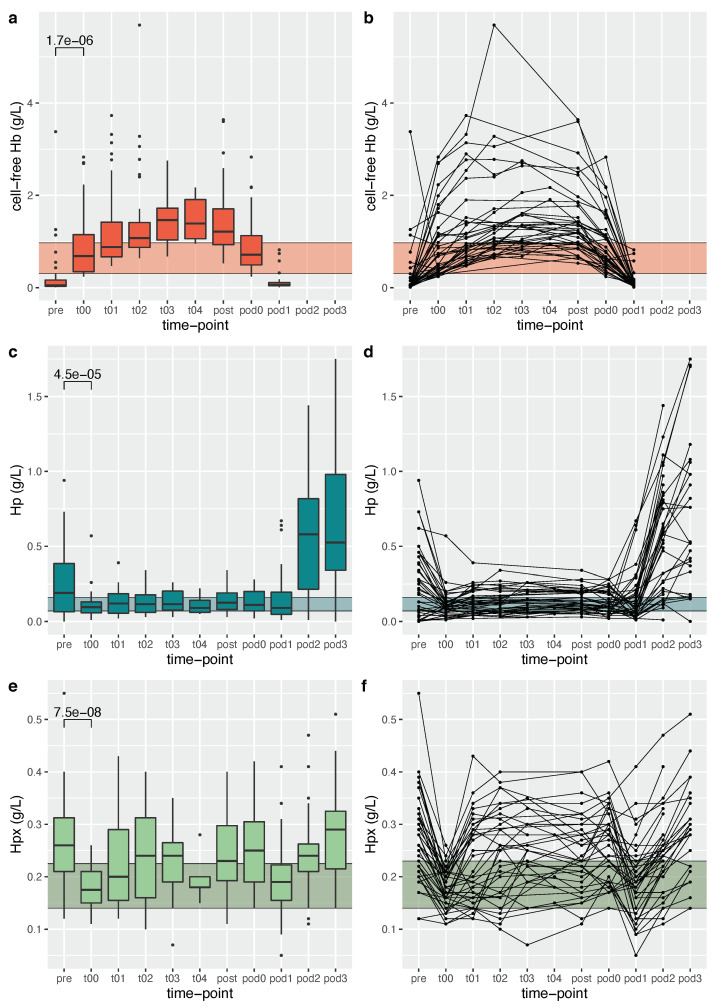
Aggregated (**a**,**c**,**e**) and individual (**b**,**d**,**f**) data on cell-free hemoglobin (**a**,**b**), haptoglobin (**c**,**d**) and hemopexin (**e**,**f**) concentrations during the study period. The shaded area represents the interquartile range of concentrations of respective protein in prime solution. Hb = hemoglobin, Hp = haptoglobin, Hpx = hemopexin, pre = preoperative sampling, t00–t04 = hourly sampling during CPB circulation, post = sampling after bypass separation, pod0 = admission to pediatric intensive care unit after surgery, pod1–3 = postoperative day 1–3.

Plasma concentrations of cell-free hemoglobin increased significantly at the start of bypass (t00) to a median concentration of 0.69 g/L (IQR 0.35–1.15) compared to a preoperative (pre) median concentration of 0.05 g/L (IQR 0.03–0.17; *p* < 0.001 for comparison) and remained elevated throughout surgery (Figure 2). Cell-free hemoglobin concentrations decreased rapidly after separation from bypass and were completely cleared from the circulation at postoperative day (pod)1.

**Figure 3:**
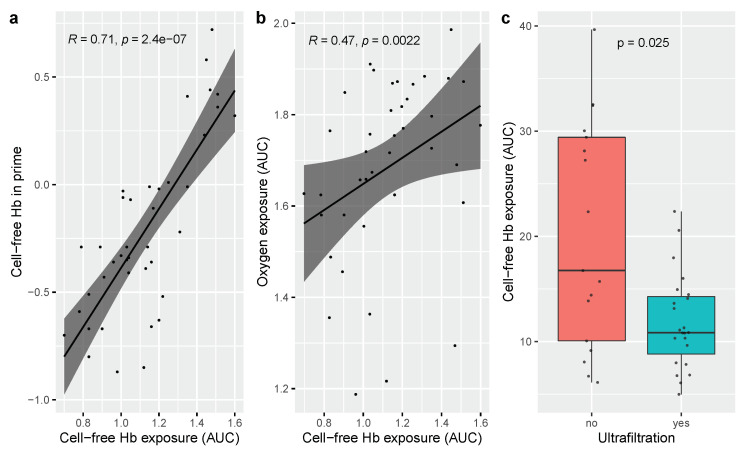
(**a**) Exposure to cell-free hemoglobin quantified as AUC plotted against concentration of cell-free hemoglobin in prime solution. Both parameters are log${}_{10}$-transformed given the nonparametric distribution. Shaded area represents 95% confidence interval for the regression model; (**b**) exposure to cell-free hemoglobin quantified as AUC plotted against oxygen exposure during cardiopulmonary bypass circulation. Both parameters are log${}_{10}$-transformed given the nonparametric distribution. Shaded area represents 95% confidence interval for the regression model; (**c**) exposure to cell-free hemoglobin quantified as AUC grouped by absence or presence of ultrafiltration during bypass circulation. Hb = hemoglobin, AUC = area under curve.

Concentration of cell-free hemoglobin in blood prime solution, volume of RBCs added to the circuit and oxygen exposure as determined by AUC were positively correlated with exposure to cell-free hemoglobin determined as area under curve (AUC) in univariable analysis (Figure 3). The presence of ultrafiltration was negatively correlated with exposure to cell-free hemoglobin in univariable analysis, Figure 3. Age of blood product in prime solution, time on bypass, vacuum-assisted venous drainage and RBC transfusion in mL/kg during surgery were not associated with exposure (AUC) to cell-free hemoglobin. When assessed in a multivariable analysis, concentration of cell-free hemoglobin in the blood prime solution was the main determinant of exposure (AUC) to cell-free hemoglobin. Univariable and multivariable relationships between evaluated variables and exposure (AUC) to cell-free hemoglobin are given in Table 3.

### 3.2. Determinants of Scavenger Protein Resources during Surgery

Fresh frozen plasma (FFP) was added to the blood prime solution at volumes of 3–22 mL/kg. Median concentration of haptoglobin in blood prime solution was 0.11 g/L (IQR 0.07–0.16). Median concentration of hemopexin in blood prime solution was 0.17 g/L (IQR 0.14–0.23).

Circulating haptoglobin concentrations decreased significantly at the start of bypass circulation (t00) to a median haptoglobin concentration of 0.10 g/L (IQR 0.06–0.13) compared to a preoperative endogenous haptoglobin median concentration of 0.19 g/L (IQR 0.07–0.39, *p* < 0.001 for comparison). Circulating hemopexin concentrations decreased significantly at the start of bypass (t00) to a median hemopexin concentration of 0.18 g/L (IQR 0.15–0.21) compared to a preoperative endogenous hemopexin median concentration of 0.26 g/L (IQR 0.21–0.31 g/L, *p* < 0.001 for comparison). Haptoglobin and hemopexin concentrations throughout the evaluated time period are illustrated in Figure 2.

Haptoglobin and hemopexin concentrations at the start of bypass circulation were positively correlated with corresponding protein concentrations in blood prime solution and preoperative circulating concentrations in multivariable analysis (*p* < 0.001 and *p* < 0.001, respectively). Volume of FFP in blood prime solution was not correlated to haptoglobin and hemopexin concentrations at bypass start in a univariable analysis.

Plasma concentrations of haptoglobin and hemopexin at pediatric intensive care unit (PICU) admission were positively correlated to the corresponding scavenger protein concentrations at bypass start when evaluated in a multivariable model including protein concentrations at bypass start, intraoperative FFP transfusion and exposure to cell-free hemoglobin during surgery (*p* < 0.001 for haptoglobin and *p* = 0.01 for hemopexin). Intraoperatively administered FFP had a minor, but statistically significant, positive influence on haptoglobin and hemopexin concentrations at PICU admission when evaluated in univariable analysis (*p* = 0.003 for haptoglobin and *p* < 0.001 for hemopexin), and for hemopexin, also when incorporated in a multivariable model (*p* < 0.001).

## 4. Discussion

This manuscript reports on cell-free hemoglobin exposure and scavenger protein concentrations during neonatal open-heart surgery on cardiopulmonary bypass. We observed that the concentration of cell-free hemoglobin in the blood prime solution was the strongest predictor of exposure to cell-free hemoglobin. The dynamics of cell-free hemoglobin concentrations over time were similar between subjects, but the magnitude of the absolute levels was already defined at bypass start. Secondly, cell-free hemoglobin scavenger capacity during bypass surgery as determined by plasma concentrations of haptoglobin and hemopexin was to a large degree modified by concentrations of the corresponding proteins in the prime solution.

The origins of cell-free hemoglobin in adult cardiopulmonary bypass surgery are commonly attributed to cardiotomy suction and passage of RBCs through the bypass circuit [9,10]. The demand for a blood primed circuit in neonatal open-heart surgery given the 1:1 relationship between neonatal blood volume and circuit volume, and the, in our study, ubiquitous need for intraoperative RBC transfusions at volumes ranging from 16–126 mL/kg, presents two additional sources of circulating cell-free hemoglobin. From our data, we conclude that the blood prime solution is crucial in determining the neonate’s exposure to cell-free hemoglobin and that the influence of cell-free hemoglobin concentrations in blood prime solution exceeds the potential impact of other intraoperative factors, e.g., prolonged bypass time or vacuum-assisted venous drainage. Interestingly, we were able to corroborate the previously reported correlation between oxygen exposure and cell-free hemoglobin concentrations in univariable analysis, but not in a subsequent multivariable analysis [11]. Neither the cohorts nor the methodology are similar between the studies making comparisons difficult, but we find the concept of oxygen stress induced hemolysis intriguing and worthy of further exploration. Transfusion of red blood cells during neonatal cardiac and non-cardiac surgery is a common procedure associated with worse outcome [12,13] and, of special relevance in a neonatal cardiac cohort, presents a risk of alloimmunization. The use of asanguineous prime solution and transfusion-free open-heart surgery has proven feasible in infants weighing <7 kg undergoing low mortality risk procedures [14].

Normal concentrations of circulating cell-free hemoglobin in healthy adults are estimated to <0.05 g/L [15], i.e., approximately 1/20 of the measured maximal concentrations in our study. Notably, the measured concentrations of cell-free hemoglobin in our cohort exceeded concentrations previously associated with injury. Prior research states a five-fold increased risk of postoperative acute kidney injury in pediatric open-heart surgery with cell-free hemoglobin concentrations ≥1 g/L persisting two hours from separation from bypass [7]. In our material, 16/40 neonates passed that threshold at PICU admission, which is a comparable time-point. Endothelial dysfunction, a proposed consequence of endothelial exposure to cell-free hemoglobin, is described at circulating concentrations of 0.097 g/L in patients with sickle cell anemia [16].

The time of maximal exposure to cell-free hemoglobin coincides with low circulating concentrations of haptoglobin, an observation in concordance with previous reports on scavenger protein concentrations during pediatric cardiac surgery [5,6]. The limited endogenous scavenger protein supplies in neonates [17], accentuated by the low concentrations of scavenger proteins in blood prime solution, results in a state of scavenger resource insufficiency at a time when most required. The binding of cell-free hemoglobin to haptoglobin is immediate, strong and estimated to occur at a binding ratio between cell-free hemoglobin and haptoglobin of 0.75 on a mass basis [15]. In our material, the absolute majority of participants presented with overwhelmed scavenging resources already at bypass start.

The use of ultrafiltration during CPB circulation was associated with a lower exposure to cell-free hemoglobin in univariable analysis. Unfortunately, there was a significant covariance between high concentrations of cell-free hemoglobin in prime solution and the absence of ultrafiltration, i.e., the neonates exposed to high concentrations of cell-free hemoglobin in blood prime solution were less likely to have a hemofilter incorporated in the bypass circuit. This might explain the lack of significance when ultrafiltration was incorporated in a multivariable model.

We acknowledge that the very high concentrations of cell-free hemoglobin (range 0.13–5.28 g/L) in some of the blood prime solutions might skew the result and obscure the influence of previously described predictors of cell-free hemoglobin exposure. We have performed the same regression analyses using a subgroup of neonates with low (≤0.5 g/L) concentrations of cell-free hemoglobin in prime solution, but, given the low number of patients (*n* = 20), these regression models were not conclusive. The evaluated sample from blood prime solution was obtained immediately before the start of bypass circulation. Hence, we cannot conclude if the almost 50-fold difference in prime solution concentration of cell-free hemoglobin represents a difference in concentrations in the packed RBC per se, or if it stems from the procedure of preparing the bypass circuit.

All neonates received irradiated, leukocyte-filtered blood in the blood prime solution. Current clinical procedure aims to reduce the risk of RBC storage lesions by using blood with as short a shelf-life as possible, which in our study translates to a median of three days (range 2–8 days). We did not observe any impact of prolonged storage of RBCs on the neonate’s exposure to cell-free hemoglobin but recognize that all packed RBCs used in prime solution had a very short storage time, and that the ability to detect potential harm from extended storage in this setting is limited.

The data presented in this article stem from a single-center prospective observational cohort study with a limited number of participants. The single-center design warrants caution when interpreting and extending our results into other clinical contexts. The study results are limited by a small sample size and the presence of covariance between evaluated variables. Moreover, we do not, in this study, address any possible clinically relevant adverse consequences of excess hemolysis. It is our understanding that prior research supports the need for an increased vigilance on the effects of circulating cell-free hemoglobin and its clearance in a neonatal cardiac surgery cohort. The correlation between hemolysis and postoperative kidney injury is well-described in pediatric and adult cardiac surgery on bypass circulation [7,8,18]. Secondly, the depletion of nitric oxide might influence microcirculation and endothelial function [19,20]. Lastly, newborns with a cCDH present with a prenatal dysmaturation of the brain [21,22], possibly rendering the brain white matter more susceptible to systemic oxidative and inflammatory stressors than expected given their gestational age [23,24].

A thorough knowledge of the influence of blood prime solution on the subsequent exposure to cell-free hemoglobin and scavenger resources is highly important when assessing possible means to mitigate the effects of circulating cell-free hemoglobin in a vulnerable cohort. The necessity of added red blood cells in neonatal bypass circulation is undisputed, whereas strategies should be developed to keep the concentrations of cell-free hemoglobin in blood prime solution as low as possible. The observation of the profound influence of cell-free hemoglobin and scavenger protein concentrations in blood prime solution enables novel prospects for minimizing cell-free hemoglobin exposure and optimizing scavenger protein resources. Pharmaceutical measures—preventing cell-free hemoglobin toxicity by administered haptoglobin or minimizing the effects of cell-free hemoglobin by administered antioxidants—have been evaluated in preclinical studies [25,26,27]. Innovative technologies, e.g., molecular imprinting technology [28] or the use of high-cutoff membranes [29], might be applicable to minimize concentrations of cell-free hemoglobin in blood prime solution before the circuit is connected to the neonate. Still, the pathophysiology of excess hemolysis in this cohort needs to be further delineated to enable properly designed treatment trials.

## 5. Conclusions

Cell-free hemoglobin concentration in blood prime solution is the main determinant of cell-free hemoglobin exposure during cardiopulmonary bypass circulation in the neonate.

## Figures and Tables

**Table 1:** Clinical characteristics of included study participants. RACHS = Risk Adjustment for Congenital Heart Surgery, IQR = inter-quartile range.

Clinical Characteristics	
Sex (male/female) (*n*)	25/15
Birthweight (gm) (mean ± SD)	3436 ± 326
Gestational age at birth (wk) (mean (min–max))	39 + 4 (37 + 6–42 + 1)
Biventricular repair (*n*)	32/40
Palliative procedure	
with prior arch obstruction (*n*)	3/40
without prior arch obstruction (*n*)	5/40
RACHS 1 (median (min–max))	4 (3–6)
Postnatal age at surgery (d) (median (IQR))	5 (4–7)

**Table 2:** Evaluated predictors of cell-free hemoglobin exposure and scavenger protein resources. IQR = inter-quartile range, RBC = red blood cell, FFP = fresh frozen plasma, AUC = area under curve.

Evaluated Predictor	
Age of blood in prime solution (d) (median (IQR))	3 (2–4)
RBC volume in prime solution (mL/kg) (median (IQR))	49 (43–56)
FFP volume in prime solution (mL/kg) (median (IQR))	12 (10–18)
Time on cardiopulmonary bypass (min) (median (IQR))	182 (142–208)
Ultrafiltration (*n*)	23/40
Vacuum-assisted venous drainage (*n*)	11/40
Oxygen exposure during bypass (AUC) (median(IQR))	52.8 (39.9–68.8)
Intraoperative transfusion RBC (mL/kg) (median (IQR))	52 (37–73)
Intraoperative transfusion FFP (mL/kg) (median (IQR))	34 (14–51)

**Table 3:** Evaluation of potential determinants of cell-free hemoglobin exposure during neonatal cardiopulmonary bypass by linear regression analysis. Values for β (95% CI) in bold denote statistically significant relationships (*p* < 0.05). Original data for evaluated determinants can be found in Table 2. CI = confidence interval, RBC = red blood cell, Hb = hemoglobin, AUC = area under curve, VAVD = vacuum-assisted venous drainage.

Variable	Univariable Analysis β (95% CI)	Multivariable Analysis β (95% CI)
Storage length of RBC unit in prime (d)	−0.30 (−2.14–1.53)	
Cell-free Hb conc in prime (g/L)	**8.50 (6.68–10.31)**	**8.11 (6.09–10.13)**
RBC volume in prime (mL/kg)	**0.39 (0.12–0.66)**	0.01 (−0.04–0.06)
Time on bypass (min)	0.02 (−0.03–0.06)	
Oxygen exposure during bypass (AUC)	**0.19 (0.09–0.29)**	0.07 (−0.003–0.14)
Ultrafiltration, yes = 1	**−8.30 (−13.15—3.44)**	−0.44 (−3.53–2.65)
VAVD, yes = 1	−2.91 (−8.96–3.13)	
Intraop RBC transfusion (mL/kg)	0.08 (−0.02–0.18)	

## Data Availability

The data presented in this study are available on request from the corresponding author. The data are not publicly available due to patient confidentiality.

## Abbreviations

The following abbreviations are used in this manuscript:

AUC	Area under curve
cCHD	Critical congenital heart defect
CPB	Cardiopulmonary bypass
FFP	Fresh frozen plasma
Hb	Hemoglobin
PICU	Pediatric intensive care unit
pod	Postoperative day
RBC	Red blood cell
VAVD	Vacuum-assisted venous drainage
